# Association of Family History with the Development of Breast Cancer: A Cohort Study of 129,374 Women in KoGES Data

**DOI:** 10.3390/ijerph18126409

**Published:** 2021-06-13

**Authors:** Hyo Geun Choi, Jung Ho Park, Yeon Ju Choi, Yong Joon Suh

**Affiliations:** 1 Department of Otorhinolaryngology-Head & Neck Surgery, Hallym University Sacred Heart Hospital, Anyang 14068, Korea; hgchoi@hallym.or.kr; 2 Department of Breast and Endocrine Surgery, Hallym University Sacred Heart Hospital, Anyang 14068, Korea; ringri@hallym.or.kr; 3 Research Cooperation Center, Hallym University, Chuncheon 24252, Korea; cyju2469@gmail.com

**Keywords:** breast cancer, family history, epidemiology, genetic predisposition, environment

## Abstract

Background: Breast cancer is the most common cancer among women. The Korean Genome and Epidemiology Study (KoGES) is a large cohort study that is available to the public. Using this large cohort study, we aimed to unravel the relationship between breast cancer development and a family history of breast cancer in Korea. Methods: This cohort study relied on data from the KoGES from 2001 through 2013. A total of 211,725 participants were screened. Of these, 129,374 women were evaluated. They were divided into two groups, including participants with and without breast cancer. A logistic regression model was used to retrospectively analyze the odds ratio of breast cancer history in families of women with and without breast cancer. Results: Of 129,374 women, 981 had breast cancer. The breast cancer group had more mothers and siblings with histories of breast cancer (*p* < 0.001). A history of breast cancer in the participant’s mother resulted in an odds ratio of 3.12 (1.75–5.59), and a history of breast cancer in the participant’s sibling resulted in an odds ratio of 2.63 (1.85–3.74). There was no interaction between the history of maternal breast cancer and the history of sibling breast cancer. Based on the subgroup analysis, family history was a stronger factor in premenopausal women than in menopausal and postmenopausal women. Conclusions: A family history of breast cancer is a significant risk factor for breast cancer in Korea. Premenopausal women with a maternal history of breast cancer are of particular concern. Intensive screening and risk-reducing strategies should be considered for this vulnerable subpopulation.

## 1. Introduction

Breast cancer is the most common cancer among women [1,2]. The incidence of breast cancer in Korea was at 5848 cases in 2000 [3]. The incidence rate of breast cancer in Korea is steadily increasing even though it is still lower than the rates in Western countries [3,4,5]. Since 2016, over 20,000 individuals per year have been diagnosed with breast cancer in Korea. The number of breast cancer cases in the United States rises with increasing patient age [1,2]. In Korea, the peak is observed at 40–49 years of age and decreases after the age of 50 [3].

The well-known risk factors for breast cancer include environmental exposure, factors related to reproduction or pregnancy, and lifestyle factors such as diet, smoking, or drinking [6,7]. Approximately 30–50% of cases are caused by these factors [8,9]. Genetic predisposition accounts for 5–10% of breast cancer [10]. However, the remaining 40–65% of cases are caused by unknown factors related to emerging areas, such as gene-gene associations and gene-environment interactions [11].

Familial breast cancer accounts for approximately 20–30% of breast cancer [8]. Family history collected during preventive care visits is defined as first- and second-degree family history [12]. First-degree family history includes parents, siblings, and children. Second-degree family history includes grandparents, aunts, uncles, grandchildren, nieces, nephews, and half siblings. Family history can identify individuals who should be referred for genetic counseling and testing, which causes considerable anxiety in women [13,14,15]. Women with a family history of breast cancer can overcome psychological distress by receiving appropriate supportive counselling [16].

The risk of breast cancer is increased up to 5.7 times in individuals with first-degree relatives who have a history of breast cancer and approximately two times in individuals with any first-degree or second-degree relatives with a history of breast cancer [17,18,19,20,21,22]. Because family history involves both genetic predisposition and environment, only a part of familial breast cancer is due to inherited genetic alterations. Inherited breast cancer is generally considered to be caused by high-penetrance BRCA 1/2 mutations [16]. Although the frequency of BRCA 1/2 mutations in the population is low, the presence of mutations in these genes can cause breast cancer with high penetrance [23]. Therefore, these mutations are frequently observed in patients with familial breast cancer.

Researchers reported a 6–9% incidence of familial breast cancer in a case-control study with approximately 3000 pairs [24]. However, the sample size was still too small to determine the association of breast cancer with family history. Recently, big data have been made available to the public, which has made large-scale research possible. The Korean government (National Research Institute of Health, Centers for Disease Control and Prevention, and the Ministry of Health and Welfare) initiated a large cohort study, which was called the Korean Genome and Epidemiology Study (KoGES) [25]. These data completed the quality control process [25]. Researchers can access these epidemiological data.

In this study, we aimed to determine the relationship between breast cancer and family history in Korea using data from the KoGES. We could perform in-depth analyses because KoGES, as an umbrella trial, has six cohorts. To the best of our knowledge, this is the first investigation of a large cohort of participants with a family history of breast cancer in Korea.

## 2. Materials and Methods

### 2.1. Study Design

**Figure 1:**
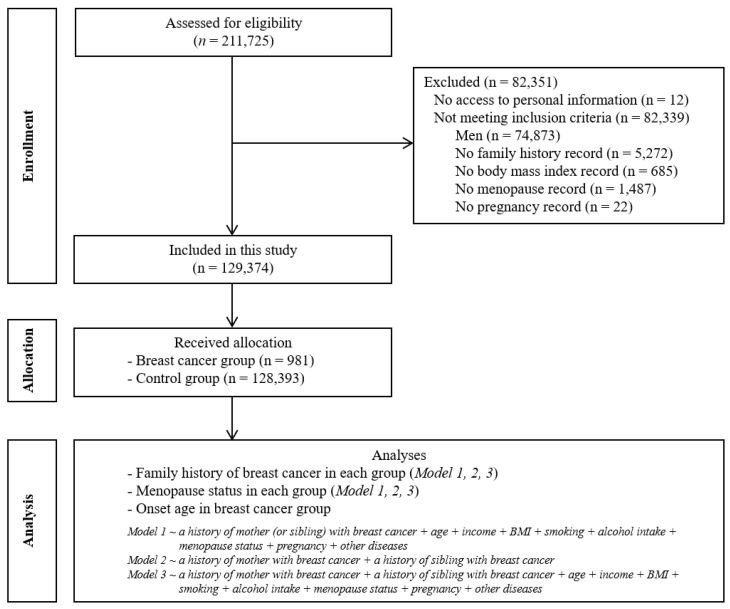
Flow diagram depicting the study design.

This cohort study relied on data from the KoGES from 2001 through 2013. A detailed description of these data is provided in a previous study [20]. A total of 211,725 participants were screened. Of these, we excluded men (n = 74,873) and participants who had no family history records (n = 5272) or records of body mass index (BMI) (n = 685), menopause (n = 1487), or pregnancy (n = 22) (Figure 1). Then, a total of 129,374 women were evaluated. Survey participants ranged from 40 to 91 years of age. Cancer incidence was identified by the Korea Central Cancer Registry. They were divided into two groups of participants, those with and without breast cancer.

### 2.2. Data Survey

Trained interviewers asked participants about their disease history of breast cancer and their age at the time of diagnosis. Participants were also asked about their family history of breast cancer and family members’ ages at the time of diagnosis. Anthropometric and clinical measurements were obtained from the KoGES consortium. In the present study, we categorized family histories of breast cancer into groups of mothers and siblings (sisters or brothers). Monthly household incomes were categorized into four groups, including no information, lowest (less than $1500), middle ($1500–$3000), and highest (more than $3000). Each participant had descriptive records of menopause status, pregnancy, and other disease history such as hypertension, diabetes mellitus, and hyperlipidemia. Obesity was measured by BMI (kg/m^2^), using height and weight as continuous variables. Smoking duration was calculated as pack-years and the consumption of alcohol was measured as the mean consumption (g/day), using the frequency and the type of alcohol.

### 2.3. Statistics

The chi-square test was used to compare the rates of sex, income, other disease history, menopause, pregnancy, and family history of breast cancer between the breast cancer group and the control group. Independent *t*-tests were used to compare age, BMI, smoking duration, and alcohol consumption. A logistic regression model was used to analyze the odds ratio of a family history of breast cancer based on breast cancer occurrence as a dependent variable. In the crude model, only family history of breast cancer was used as an independent variable. Model 1 was adjusted for age, income, BMI, smoking, alcohol intake, menopause status, pregnancy, and other diseases. Model 2 was adjusted for family histories of mothers and family histories of siblings with breast cancer. Model 3 was adjusted for the variables from models 2 and 3. Additionally, we analyzed the interaction in model 4, which was adjusted for a history of maternal breast cancer, history of sibling breast cancer, and history of maternal and sibling breast cancer. The adjusted odds ratios and 95% confidence intervals were calculated from the final model. Two-tailed analyses were conducted, and *p* values less than 0.05 indicated significance. The results were statistically analyzed using SPSS 22.0 (IBM, Armonk, NY, USA).

## 3. Results

From a total of 129,374 women, 981 women had experienced breast cancer. They were categorized as the breast cancer group. Therefore, 128,393 women without breast cancer were categorized as the control group. The average age in the breast cancer and control groups was 54.2 ± 7.5 and 53.4 ± 8.5 years, respectively (Table 1). There were no significant differences in other diseases (hypertension, diabetes, and dyslipidemia) between the two groups. More women in the breast cancer group had family histories of mothers and siblings with breast cancer (*p* < 0.001).

In model 1, a family history of a mother with breast cancer resulted in an odds ratio of 3.21 (1.80–5.74), and a family history of a sibling with breast cancer resulted in an odds ratio of 2.66 (1.87–3.78). In model 2, a family history of a mother with breast cancer resulted in an odds ratio of 2.78 (1.56–4.94), and a family history of a sibling with breast cancer resulted in an odds ratio of 2.86 (2.02–4.07). In model 3, a family history of a mother with breast cancer resulted in an odds ratio of 3.12 (1.75–5.59), and a family history of a sibling with breast cancer resulted in an odds ratio of 2.63 (1.85–3.74) (Table 2). There was no interaction between a family history of a mother with breast cancer and a family history of a sibling with breast cancer (Table 3).

In the subgroup analysis, participants were divided into groups of 80,583 women who had experienced menopause and 48,791 women who had not experienced menopause (Table 4). In women who had experienced menopause, a family history of a mother with breast cancer resulted in an odds ratio of 2.50 (1.17–5.35), and a family history of a sibling with breast cancer resulted in an odds ratio of 2.53 (1.72–3.71). In women who had not experienced menopause, a family history of a mother with breast cancer resulted in an odds ratio of 4.93 (1.99–12.20), and a family history of a sibling with breast cancer resulted in an odds ratio of 3.13 (1.27–7.72). Based on the subgroup analysis, family history was a stronger factor in women who had not experienced menopause than in those who had experienced menopause.

Of 981 women, nine participants were excluded from the subgroup analysis due to missing values for age. Thus, in 972 women with breast cancer, family history and age were re-evaluated with a cut-off of 50 years (Table 5). A greater number of women with a family history of relatives with breast cancer were younger than 50 years old, although the difference was not significant.

## 4. Discussion

In the present study, we performed a risk calculation of family history based on a Korean nationwide public registry database. By analyzing this public database, meaningful family histories were identified in patients with breast cancer. The odds ratios were 3.12 for family history of a mother with breast cancer and 2.63 for family history of a sibling with breast cancer. There was no interaction between history of maternal breast cancer and history of sibling breast cancer. Furthermore, these family histories were more influential in premenopausal women. Only among women with breast cancer were more family histories observed in the group with onset ages less than 50 years, although the results were not statistically significant.

To the best of our knowledge, this is the first investigation of a large cohort of participants with a family history of breast cancer in Korea. After adjustment of multiple environmental risk factors, our study showed that family history was a strong risk factor for breast cancer in a large cohort of Asian women. Particularly, premenopausal women with a maternal history of breast cancer were the most vulnerable group, of which the odds ratio was almost five times higher than the control group. Age, BMI, pregnancy, menopause, other disease, smoking, alcohol consumption, or income could be related to the risk of breast cancer, according to previous studies [2,26,27]. However, some studies did not consider these factors when calculating the risk of family history [19,21,28]. Large-scale data from epidemiologic studies are essential for determining the association between cancer and family history because familial breast cancer is relatively rare. Therefore, our calculations were statistically adjusted by these variables using big data from breast cancer and control groups. Furthermore, the findings were supported by in-depth analyses because KoGES, as an umbrella trial, has six cohorts. Various data in KoGES as an open access resource can be legitimately presented to investigators who have interests in further research derived from the current study.

Inherited breast cancer is different from familial breast cancer, which is driven not only by genes but also by interactions with the environment. However, there is a strong possibility of inherited breast cancer among familial breast cancers [29,30]. BRCA 1/2 are the representative genes assessed in inherited breast cancer, although a risk assessment should be performed for all women [18,31]. Therefore, adult women with a family history of breast cancer are eligible for BRCA 1/2 testing, although the analysis of BRCA 1/2 mutations was not available in our study due to the lack of BRCA information in the KoGES database. BRCA, as a tumor suppressor gene, repairs DNA damage and inhibits neoplasms [32]. This examination is performed using peripheral blood after genetic counseling based on the individual’s pedigree. Imaging studies and clinical data are usually needed for breast cancer screening [33]. Women with BRCA 1/2 mutations should undergo annual magnetic resonance imaging (MRI) scans between the ages of 25 and 29 years, and annual mammograms and MRI scans between the ages of 30 and 75 years [34,35]. Women harboring these mutations have a lifetime risk of 46–87% for breast cancer and 39–63% for ovarian cancer [36]. However, they can take tamoxifen or raloxifene to reduce their risk [34]. For women who have a lifetime risk ≥ 20%, for breast cancer, annual MRI scans and mammograms starting at 10 years prior to the age at which the youngest family member developed breast cancer, but not prior to the age of 25 and 30 years, respectively, are recommended even in the absence of BRCA 1/2 mutations [37]. As women with family members diagnosed with breast cancer diagnosed after 50 years of age have an average risk, an annual mammogram is recommended for these women [2,33].

The records regarding a family history of cancer have not been standardized in a structured manner [38]. However, these records should include cancer type, lineage, degree, age at diagnosis, and ethnicity [12,39]. It is recommended to update an individual’s family history of cancer every 5–10 years between 30 and 60 years of age [38,40]. The American Cancer Society (ACS) guidelines also recommend annual MRI scans plus mammograms for women with family members harboring breast cancer-associated mutations, and these women should also undergo a genetic assessment [32,41]. In the current study, the KoGES provided only the family history as pertains to an individual having a mother or sibling with breast cancer, which does not satisfy the recommended minimum record regarding a family history of cancer. Participants included in the present cohort should update their family history for future research.

The present study has some limitations. First, this study was based on the limited data provided, which is a general limitation of public data analysis. Therefore, our study could not consider covariates such as age at menarche, age at first birth, previous biopsies, or occupational exposures, which might be related to breast cancer. Moreover, we could not conduct a comprehensive investigation of first- and second-degree family histories. Second, there could be recall bias given the use of a survey to collect data, which could lead to less reliable results. Last, the present study has the usual limitations of observational studies such as detection bias and selection bias.

## 5. Conclusions

In conclusion, a family history of breast cancer is a significant risk factor for breast cancer in Korea. The identification of a family history of breast cancer can contribute to early detection in populations at risk. In particular, premenopausal women with a maternal history of breast cancer are at the highest risk. Intensive screening and risk-reducing strategies should be considered for this vulnerable subpopulation.

## Figures and Tables

**Table 1:** General characteristics of participants in the present study.

Variable	Total Participants		*p*
	Breast Cancer	Control	
Total Number (n, %)	981 (100.0)	128,393 (100.0)	
Age (year)	54.2 ± 7.5	53.4 ± 8.5	n.s
Income (n, %)			<0.001 ^†^
No information	145 (14.8)	26,826 (20.9)	
Lowest	227 (23.1)	28,348 (22.1)	
Middle	289 (29.5)	32,927 (25.6)	
Highest	320 (32.6)	40,292 (31.4)	
Hypertension (n, %)			n.s
Yes	165 (16.8)	24,417 (19.0)	
No	816 (83.2)	103,976 (81.0)	
Diabetes (n, %)			n.s
Yes	67 (6.8)	7379 (5.7)	
No	914 (93.2)	121,014 (94.3)	
Dyslipidemia (n, %)			n.s
Yes	100 (10.2)	11,011 (8.6)	
No	881 (89.8)	117,382 (91.4)	
Pregnancy (n, %)			0.001 ^†^
Yes	934 (95.2)	124,613 (97.1)	
No	47 (4.8)	3780 (2.9)	
Menopause (n, %)			<0.001 ^†^
Yes	835 (85.1)	79,748 (62.1)	
No	146 (14.9)	48,645 (37.9)	
BMI (kg/m^2^)	23.5 ± 2.9	23.8 ± 3.0	n.s
Smoking (pack-year)	0.22 ± 2.12	0.56 ± 3.57	<0.001 *
Alcohol (g/day)	0.66 ± 3.71	1.81 ± 7.75	<0.001 *
Family history of mother (n, %)			<0.001 ^†^
Breast cancer	12 (1.2)	546 (0.4)	
No breast cancer	969 (98.8)	127,847 (99.6)	
Family history of sibling (n, %)			<0.001 ^†^
Breast cancer	33 (3.4)	1519 (1.2)	
No breast cancer	948 (96.6)	126,874 (98.8)	

n.s = not significant. * Independent *t*-test. † Chi-square test.

**Table 2:** Association of family history with the development of breast cancer.

Variable	Odds Ratio of Breast Cancer							
	Crude	*p*	Model 1	*p*	Model 2	*p*	Model 3	*p*
Family History of a Mother with Breast Cancer								
Breast cancer	2.90 (1.63–5.16)	<0.001 *	3.21 (1.80–5.74)	<0.001 *	2.78 (1.56–4.94)	0.001 *	3.12 (1.75–5.59)	<0.001 *
Control	1.00		1.00		1.00		1.00	
Family history of a sibling with breast cancer								
Breast cancer	2.91 (2.05–4.13)	<0.001 *	2.66 (1.87–3.78)	<0.001 *	2.86 (2.02–4.07)	<0.001 *	2.63 (1.85–3.74)	<0.001 *
Control	1.00		1.00		1.00		1.00	

Model 1: adjusted for age, income, body mass index, smoking, alcohol consumption, menopause status, pregnancy, and other diseases including hypertension, diabetes mellitus, and dyslipidemia. Model 2: adjusted for history of a mother with breast cancer and history of a sibling with breast cancer. Model 3: adjusted for model 1 and model 2. * Logistic regression analysis.

**Table 3:** Analysis of interaction between a family history of a mother with breast cancer and a family history of a sibling with breast cancer.

Variable	Odds Ratio of Breast Cancer	*p*
	Model 4	
Family history of maternal breast cancer	2.56 (1.36–4.79)	0.003 *
Family history of sibling breast cancer	2.78 (1.94–4.00)	<0.001 *
Family history of maternal and sibling breast cancer	1.99 (0.39–10.13)	0.406

Model 4: adjusted for history of maternal breast cancer, history of sibling breast cancer, and history of maternal and sibling breast cancer. * Logistic regression analysis.

**Table 4:** Subgroup analysis of the association between breast cancer and family history according to menopause status.

Variable	Odds Ratio of Breast Cancer							
	Crude	*p*	Model 1	*p*	Model 2	*p*	Model 3	*p*
Menopause (n = 80,583)								
Family history of a mother with breast cancer								
Breast cancer	2.86 (1.35–6.09)	0.006 *	2.56 (1.20–5.47)	0.015 *	2.78 (1.30–5.91)	0.008 *	2.50 (1.17–5.35)	0.018 *
Control	1.00		1.00		1.00		1.00	
Family history of a sibling with breast cancer								
Breast cancer	2.68 (1.83–3.93)	<0.001 *	2.54 (1.73–3.73)	<0.001 *	2.66 (1.82–3.90)	<0.001 *	2.53 (1.72–3.71)	<0.001 *
Control	1.00		1.00		1.00		1.00	
No menopause (n = 48,791)								
Family history of a mother with breast cancer								
Breast cancer	5.51 (2.24–13.54)	<0.001 *	5.23 (2.12–12.89)	<0.001 *	5.18 (2.10–12.78)	<0.001 *	4.93 (1.99–12.20)	0.001 *
Control	1.00		1.00		1.00		1.00	
Family history of a sibling with breast cancer								
Breast cancer	3.42 (1.39–8.37)	0.007 *	3.36 (1.37–8.23)	0.008 *	3.17 (1.29–7.81)	0.012 *	3.13 (1.27–7.72)	0.013 *
Control	1.00		1.00		1.00		1.00	

Model 1: adjusted for age, income, body mass index, smoking, alcohol intake, menopause status, pregnancy, and other diseases including hypertension, diabetes mellitus, and dyslipidemia. Model 2: adjusted for history of a mother with breast cancer and history of a sibling with breast cancer. Model 3: adjusted for model 1 and model 2. * Logistic regression analysis.

**Table 5:** Risk calculation for family history of breast cancer according to the onset of breast cancer.

Variable	Onset of Breast Cancer		*p*
	<50 years	≥50 years	
Family history of a mother with breast cancer (n, %)			
Yes	8 (1.4)	4 (1.0)	0.771 *
No	573 (98.6)	387 (99.0)	
Family history of a sibling with breast cancer (n, %)			
Yes	19 (3.3)	14 (3.6)	0.793 ^†^
No	562 (96.7)	377 (96.4)	

* Fisher’s exact test. † Chi-square test.

## Data Availability

All the data supporting the findings of this study are available from the corresponding author upon reasonable request.
